# Nitric Oxide-Based Treatment of Poor-Grade Patients After Severe Aneurysmal Subarachnoid Hemorrhage

**DOI:** 10.1007/s12028-019-00809-1

**Published:** 2019-08-15

**Authors:** Angelika Ehlert, Jitka Starekova, Gerd Manthei, Annette Ehlert-Gamm, Joachim Flack, Marie Gessert, Joachim Gerss, Volker Hesselmann

**Affiliations:** 1 Department of Neurosurgery, Asklepios Hospital St. Georg, Lohmühlenstr. 5, 20099 Hamburg, Germany; 2 Department of Radiology, University Hospital Hamburg-Eppendorf, Martinistr. 52, 20251 Hamburg, Germany; 3 Doctor’s Office, Christinenstr. 1, 10119 Berlin, Germany; 4 Doctor’s Office, Breitenfelderstr. 7, 20251 Hamburg, Germany; 5 Department of Neurology, Asklepios Hospital St. Georg, Lohmühlenstr. 5, 20099 Hamburg, Germany; 6 Institute of Biostatistics and Clinical Research, University Hospital Münster, Schmeddingstr. 56, 48149 Münster, Germany; 7 Department of Neuroradiology, Asklepios Hospital Nord, Tangstedter Landstr. 400, 22417 Hamburg, Germany

**Keywords:** Poor-grade aneurysmal SAH, DCI, Coma, Intraventricular/intravenous nitric oxide donor, Molsidomine, Sodium nitroprusside, Vasospasm, Transcranial Doppler sonography

## Abstract

**Background:**

Patients with aneurysmal subarachnoid hemorrhage (aSAH) require close treatment in neuro intensive care units (NICUs). The treatments available to counteract secondary deterioration and delayed ischemic events remain restricted; moreover, available neuro-monitoring of comatose patients is undependable. In comatose patients, clinical signs are hidden, and timing interventions to prevent the evolution of a perfusion disorder in response to fixed ischemic brain damage remain a challenge for NICU teams. Consequently, comatose patients often suffer secondary brain infarctions. The outcomes for long-term intubated patients w/wo pupil dilatation are the worst, with only 10% surviving. We previously added two nitroxide (NO) donors to the standard treatment: continuous intravenous administration of Molsidomine in patients with mild-to-moderate aSAH and, if required as a supplement, intraventricular boluses of sodium nitroprusside (SNP) in high-risk patients to overcome the so-called NO-sink effect, which leads to vasospasm and perfusion disorders. NO boluses were guided by clinical status and promptly reversed recurrent episodes of delayed ischemic neurological deficit. In this study, we tried to translate this concept, the initiation of intraventricular NO application on top of continuous Molsidomine infusion, from awake to comatose patients who lack neurological–clinical monitoring but are primarily monitored using frequently applied transcranial Doppler (TCD).

**Methods:**

In this observational, retrospective, nonrandomized feasibility study, 18 consecutive aSAH comatose/intubated patients (Hunt and Hess IV/V with/without pupil dilatation) whose poor clinical status precluded clinical monitoring received standard neuro-intensive care, frequent TCD monitoring, continuous intravenous Molsidomine plus intraventricular SNP boluses after TCD-confirmed macrospasm during the daytime and on a fixed nighttime schedule.

**Results:**

Very likely associated with the application of SNP, which is a matter of further investigation, vasospasm-related TCD findings promptly and reliably reversed or substantially weakened (*p* < 0.0001) afterward. Delayed cerebral ischemia (DCI) occurred only during loose, low-dose or interrupted treatment (17% vs. an estimated 65% with secondary infarctions) in 17 responders. However, despite their worse initial condition, 29.4% of the responders survived (expected 10%) and four achieved Glasgow Outcome Scale Extended (GOSE) 8–6, modified Rankin Scale (mRS) 0–1 or National Institutes of Health Stroke Scale (NIHSS) 0–2.

**Conclusions:**

Even in comatose/intubated patients, TCD-guided dual-compartment administration of NO donors probably could reverse macrospasm and seems to be feasible. The number of DCI was much lower than expected in this specific subgroup, indicating that this treatment possibly provides a positive impact on outcomes. A randomized trial should verify or falsify our results.

## Introduction

Following aneurysmal subarachnoid hemorrhage (aSAH), oxygenated, high-pressure blood accumulates in the subarachnoid space [1] where it increases intracranial pressure (ICP) and evokes early brain injury (EBI) [2–4], ultra-early (< 6 h) vasospasm [5] and cortical and/or global cerebral ischemia [6, 7]. During the subsequent week(s), 70% of all-grade patients develop delayed macrospasm and an uncertain number of spasms in the microvasculature and up to 50% have delayed cerebral ischemia (DCI) with poor outcomes. After a ruptured aneurysm is fixed, this natural history of aSAH shifts therapeutic responsibility from the neurosurgical to the neuro intensive care unit (NICU) team, which has limited therapeutic choices (Nimodipine and/or induced hypertension) [8–10] while monitoring clinical status, blood and ICP, laboratory parameters and blood flow velocity by transcranial Doppler (TCD).

The exact pathomechanisms of EBI, early and/or delayed vasospasm, delayed ischemic neurological deficit (DIND) followed by DCI and poor outcomes remain debated [6]. It is, however, widely accepted that oxyhemoglobin and its degradation products, which are potent nitroxide (NO) scavengers, derail the vasodilatory activity of endothelial and neuronal NO synthases, further evoking endothelial dysfunction ([11–13], please see “Appendix”). Decreased NO availability in the arterial wall endothelium coupled with the depletion of brain NO stores [14], which affects conductive arteries and microcirculation and limits the perfusion of the cortex by triggering local vasospasm, spreading depolarizations and leading to spreading ischemia [15, 16].

Previously, an intravenous infusion of NO-donor Molsidomine [17, 18] (see “Appendix”) demonstrated a significant improvement in outcomes with lower rates of DCI [17] in all-grade aSAH patients but had no impact on macrovasospasm; its effect is thought to be on microcirculation. Additionally, in an awake female patient and guided by her clinical status, multiple clinical DCIs and radiologically confirmed vasospasms were rapidly reversed by combining intravenous Molsidomine with intraventricular sodium nitroprusside (SNP) boluses, which achieved excellent outcomes [18].

Whether an intravenous and on-demand intraventricular NO substitution can also be useful in extremely difficult to monitor, comatose, Hunt and Hess grade IV/V patients in a bedside NICU setting is addressed in this study.

## Materials and Methods

This study took place in the intensive care unit (ICU) of a maximum care hospital in 2016–2017. The design of this feasibility study is observational, nonrandomized and retrospective. The endpoints of the study were the relief of vasospasm as measured by TCD and the numbers of secondarily developed delayed brain infarctions; the latter were compared to the results expected in this subgroup. We included 18 subsequent long-term comatose patients (8 males, 10 females) aged 38–83 years (mean 59 years; SD 13 years; range 32–83 years) post-aSAH (modified Fisher IV [1]) in Hunt–Hess grades IV (without) and V (with) pupil dilatation ([19–21], definitions in “Appendix”) who did not recover after cerebrospinal fluid (CSF) drainage and treatment of the aneurysm. Including eight patients with intracerebral and one with subdural hematomas, seven patients presented with ultra-early vasospasm (< 6 h after aSAH, Table 1). All patients had poor prognoses with an expected survival rate of 10% [22, 23] and the maximal risk of developing delayed vasospasm and delayed brain infarctions. Additionally, an NO-based treatment was offered to supplement standard therapy. Verbal and subsequent written informed consent was obtained from relatives or legal guardians within 12–36 h after aSAH. This retrospective, nonrandomized feasibility study met the principles of the Declaration of Helsinki and was approved by the local ethics committee (PV5410).

**Table 1:** Patient characteristics, treatments and complications

Patients	Hunt and Hess IV/modified Fisher IV, no pupil dilatation	Hunt and Hess V/modified fisher IV, dilated pupils
Total = 18	12^a^ (1 nonresponder)	6^a^
Mean age	59.6 (range 38–83)	58.5 (range 41–80)
Gender	7 Females, 5 males	3 Females, 3 males
Aneurysm location	MCA *n* = 3, BA *n* = 3, PICA *n* = 1, ACA/A. communicans Anterior *n* = 5, ICA *n* = 2, multiple locations *n* = 2	BA *n* = 1, VA *n* = 1, PICA *n* = 1, MCA *n* = 2, ACoA *n* = 2, ICA *n* = 1, multiple location: *n* = 2
Treatment	Coil *n* = 10, Clip *n* = 2	Coil *n* = 3, Clip: *n* = 2, combined *n* = 1
Medical history	*n* = 11: Chronic obstructive pulmonary disease, arterial hypertension nephropathy, diabetes mellitus, stroke	*n* = 3: arterial hypertension, alcohol abuse
Missing segments in the circle of Willis—perfusion by a dominant vessel	1	1
Ventriculostomy	12	6
Intubated and sedated	12^b^	6
Re-bleeding	0	2
Ultra-early vasospasm	4^b^	3
Initial reanimation, intra- cerebral hemorrhage (*n* = 8, 5 surgically treated) and acute subdural hematoma (*n* = 1), ventricular fibrillation	5	6
Coiling or clipping associated infarctions	3	2
Severe complications: sinus thrombosis, type II heparin-induced thrombosis, pulmonary embolism, sepsis, acute renal failure, aspiration pneumonia, diabetes insipidus, brain edema (with craniectomy, *n* = 5)	8	7
Intraventricular hemorrhage	2	4

*ACA* anterior cerebral artery, *ACoA* anterior communicanting artery, *BA* basilar artery, *ICA* internal cerebral artery, *MCA* middle cerebral artery, *PICA* posterior inferior cerebellar artery

^a^Not due to hydrocephalus

^b^Including a nonresponder

As clinical worsening is concealed in comatose patients, currently available surrogate parameters are difficult to interpret and prone to failure [24]. Even the use of continuous TCD, electrocochleography or electroencephalogram (EEG) recordings in unconscious individuals does not replace the clinical alarm signal of DIND because their placement, sensitivity and specificity are inconsistent. The clinical relevance of findings could therefore be unclear or is often recognized only after the ischemic lesion has already occurred.

Monitoring of the comatose patients in this study included internationally recommended ICU standards, near-infrared spectroscopy (NIRS, CASMED Fore-Sight ELITE, Branford CT, USA), real-time assessment of regional cortical oxygenation and ICP monitoring. For dedicated surveillance of these unconscious patients, we frequently applied TCD screening of all major brain-supplying arteries, with special attention to the aliasing, pulsatility, flow turbulences, flow velocities, a spectral broadening and shape changes, especially the appearance of tardus and parvus waves.

After the ruptured aneurysm was secured, each patient started an oral (6 × 60 mg) or intravenous (1–2 mg/h) Nimodipine [9, 10] regimen according to accepted standards.

Mean arterial blood pressure (MAP) was kept at > 65 mmHg when there was no evidence of vasospasm on diagnostic imaging or TCD and at 80–105 mmHg (zeroing at cardiac atrium) when vasospasm was detected. The estimated degree of vasospasm, the presence or absence of suspected disorders of microcirculation, general medical conditions, preexisting parenchymal bleeding or coiling and clipping-related infarctions, the amount of vasopressors (if) needed and the possible impact of Nimodipine on systemic blood pressure were considered. Molsidomine was started intravenously at 1.6 mg/h (2 ampoules/20 mg diluted in 50 ml saline and started at 2 ml/h) within 12–36 h after aSAH. The Molsidomine dose was increased in a stepwise manner (1.6 mg/h) every 3–4 h to achieve a dose of 16 mg/h within 2–3 days [17]. No more than 500 µg/h of noradrenaline was needed to keep MAP > 65 mmHg. During episodes of infection, low cardiac output, or isoflurane administration, noradrenaline was increased (900–8350 µg/h) with occasional adjustment of the Molsidomine dose. At the end of therapy, in parallel with Nimodipine, the Molsidomine dose was tapered for 5–8 days.

TCD was used to detect vasospasm in conductive arteries. It was performed at least three times during daytime and on-demand during periods of increased risk of vasospasm. Macrospasm was assumed when (a) the mean flow exceeded 150 cm/s (probable), 190 cm/s (severe) or 290 cm/s (critical) in the middle cerebral artery and the anterior cerebral arteries); (b) when it exceeded > 120 cm/s (severe) in the vertebral/basilar artery; and (c) when significant changes were observed in TCD flow signals (appearance of an alias phenomenon, increase in mean velocity with spectral broadening, elevated pulsatility index, turbulent flow, tardus and parvus waves). Mean flows in stenoses prior to tardus and parvus segments were extrapolated > 350 cm/s. Caution was advised for any sudden flow acceleration or a Lindegaard’s index > 3 [25, 26]. Almost all TCD measurements were performed by the same examiner to avoid interobserver variability.

If despite the general treatment, including the induction of hypertension, a TCD-confirmed severe macrospasm occurred, a bedside SNP bolus was applied via an external ventricular drain (EVD). Each bolus contained 2–10 mg of SNP (mean 5.6 mg, SD 2.5 mg) [18]. The volume of the SNP bolus was adjusted for the dead space of the catheter (3 ml). If the ICP prior to SNP was elevated (up to 20–22 mmHg), the concentration of the SNP was doubled or quadrupled to reduce the volume burden, and 5 ml of CSF was removed; each application was applied on a case-by-case basis. No SNP was applied if the baseline ICP was higher than this limit. After the SNP bolus as administered, the EVD was closed for < 5 min, and ICP and MAP were monitored. If ICP reached > 30 mmHg, the EVD was opened to release a few milliliters of CSF/SNP mixture until the ICP returned to a level under 20 mmHg. No post-SNP elevation in ICP persisted, and all patients returned to baseline limits after a few minutes. ICP increases were frequently accompanied by tachycardia. The cranial computed tomography controls showed no associated brain edema.

After saturation with Molsidomine, each SNP bolus (except in the case of the nonresponder) led to a generally enormous increase in systemic blood pressure that was interpreted as a brainstem reflex on intracranial/intracerebral vasodilation [27]. No bradycardia occurred, making a Cushing reflex implausible.

Control TCDs were performed after SNP administration once MAP reached values prior to application again (within 20–30 min) to exclude an impact of systemic hypertension. Therapeutic success was assumed if a normalization of TCD values and regular signals (re)occurred (Fig. 2), because a high-grade macrospasm commonly persists for a much longer interval than 30–60 min.

The presence of serious, recurrent vasospasm on TCD prompted first an SNP bolus and, if advisable, a computed tomography angiography (CTA) and/or CT perfusion (CTP) study to quantify the findings (*n* = 28). If advisable and achievable in a timely manner, digital subtractive angiography (DSA) was performed in the preparation for an intra-arterial administration of Nimodipine (chemical vasospasmolysis: 18 events in 10 patients, *n* = 6 in the nonresponder). A few patients received SNP bolus(es) just prior to CTA, CTP diagnostics or DSA, and this allowed an assessment of effects.

CT was performed on days 1, 3, 7 and 14 after aSAH, before discharge, on-demand, and 24 h after each neurosurgical or neuroradiological intervention. Supplementary CTP, CTA and DSA were used to diagnose vasospasm and perfusion disorders and as a possible transluminal intervention. As the patients were critically ill, imaging was performed only when clinically justifiable with a preference for the less risky CT than magnetic resonance imaging (MRI). In surviving patients, MRI was performed before discharge and during follow-up.

Findings were independently assessed for vasospasm and hypodensities by a blinded, experienced neuroradiologist who was not a member of the study team. The neuroradiological assessments included an evaluation of the threshold-based interpretation of CT perfusion using mean transit time (MTT) and cerebral blood flow/volume (CBF/CBV) measurements. Pathological values were defined according to [28]: MTT values > 6.4 s, CBF and CBV values < 39.3 and < 4.4 cm 3/100 g, respectively, in comparison with the degree of reduction in vascular caliber measured by a caliper on DSA.

Additionally, the impact on microcirculation was assessed by estimating the flooding and washing out times of contrast media in affected vessels. A comparison of arterial stenosis findings between CTA and conventional DSA was performed using an electronic caliper. If applicable, perfusion disorders and their resolution were examined by comparing them prior to post-SNP application. Newly arisen hypodensities observed on CT were evaluated for possible classification (postoperative, other causes, related to spasm) by an experienced neuroradiologist.

Initially, intraventricular TCD-guided SNP boluses were administered in two patients during the daytime. A normalization of malignant 4-vessel vasospasm was achieved after the administration of repeated boluses of SNP. However, vasospasm recurred during the following nights, and both patients died with massive brain infarctions. Consequently, SNP administration at fixed intervals was introduced during the nights when TCD was unavailable.

**Fig. 1:**
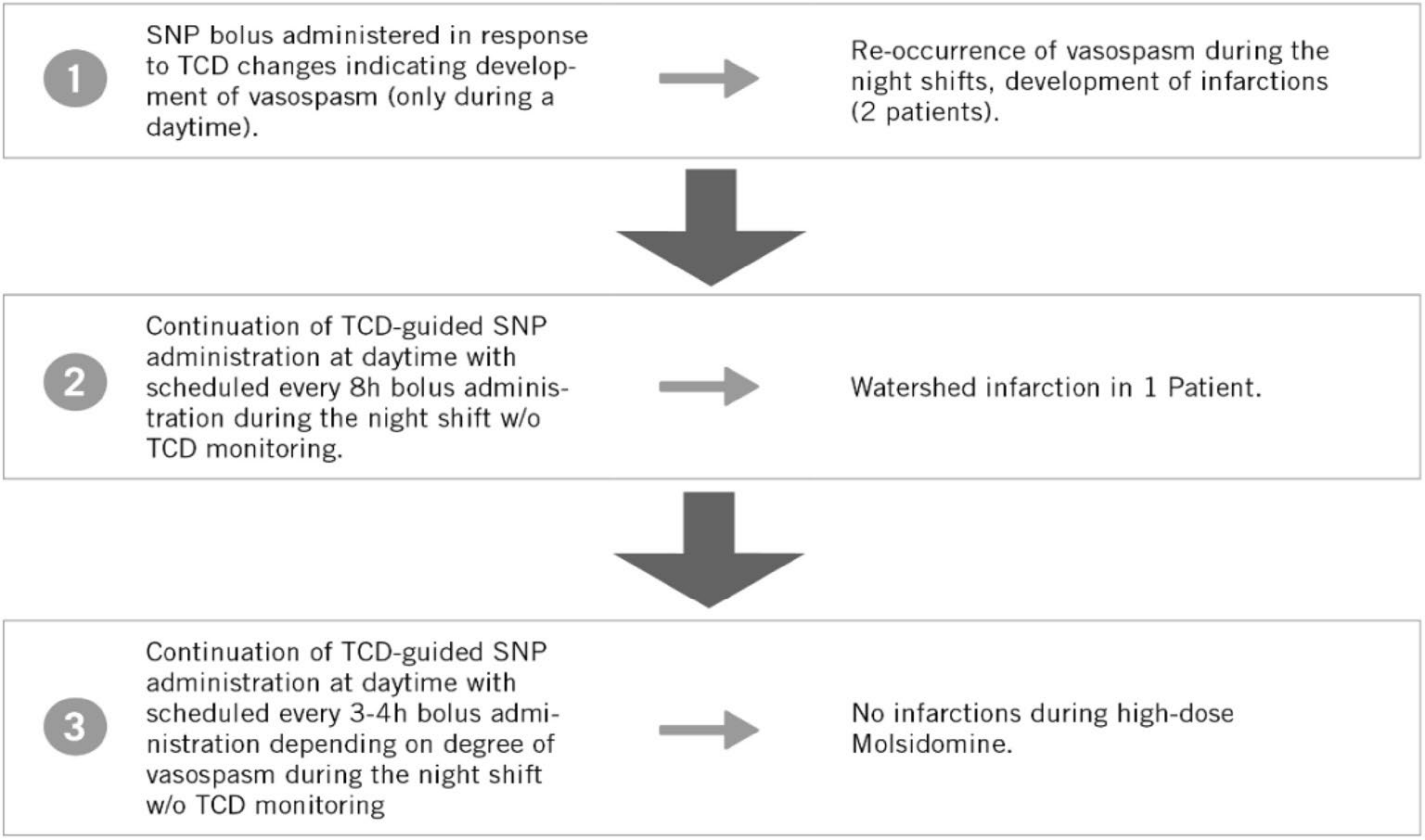
Response-guided evolution of decision tree for SNP administration

First, nocturnal bridging was used every 8 h, but the occurrence of a small watershed infarction in one patient prompted a change to SNP boluses every 3–4 h (Fig. 1). Subsequently, no further secondary brain ischemia occurred, except for treatment interruptions in two patients.

For cumulative daily quantities > 50 mg SNP, a prophylactic intravenous administration of sodium thiosulfate (50 mg/kg of body weight) was considered to minimize the risk of cyanide poisoning.

To control a hypertensive crisis evoked by SNP-induced intracerebral vasodilatation, 40–360 mg of Urapidil and/or 50–300 mg of propofol were used, and accompanying tachycardia or tachyarrhythmia was reverted after Metoprolol. To limit nausea and vomiting after SNP bolus administration, most patients required intravenous Ondansetron and the evacuation of gastric content-delaying oral Nimodipine administration.

Silver-coated ventricular catheters were replaced every 14 days to minimize the risk of ventriculitis. CSF samples were analyzed every 2 days.

Determinations of Molsidomine levels and SNP degradation products in CSF were not technically possible. Laboratory monitoring was limited to blood gas, lactate and methemoglobin levels.

The follow-up of survivors was performed by independent physicians at 6 and 18 months after discharge and included an MRI and a visit.

### Statistical Analysis

Statistical analyses were performed using SAS version 9.4 (SAS Institute Inc., USA). Beyond descriptive statistics, TCD and NIRS values were compared between pre- and post-SNP using linear mixed models, and the corresponding *p* values were calculated. The models included a subject-specific random intercept to account for repeated measurements of TCD and NIRS values in the same patient using a restricted maximum likelihood approach. Based on the fitted models, the mean differences in TCD and NIRS values between pre- and post-SNP were evaluated for significance. All results are considered exploratory, not confirmatory. *p* values are regarded as noticeable (“significant”) when *p* ≤ 0.05 without adjustment for multiple testing. An overall significance level was not determined and could not be calculated. Power analysis revealed that in our study, 17 patients with an average of 10 (pathological) measurements per patient and an expected intraclass correlation *ρ* = 0.5 provided at least 80% power to detect a clinically relevant effect of SNP on mean flow velocities and NIRS. This means that after SNP administration, mean flow velocities decreased by 13% (a measurable change in the mean between pre- and post-SNP, with a standard deviation ± 50%), and NIRS values increased by 15% (± 25%) or more.

## Results

Seventeen patients (including the nonresponder) received and tolerated well the continuous intravenous high-dose administration of Molsidomine at 16 mg/h. One patient, due to septic conditions, reached a maximum of 6.4 mg/h.

All patients developed delayed cerebral vasospasm with perfusion disturbances between the 3rd and 13th day (mean 7 days; SD 2.5 days) as diagnosed by TCD, CTA, CTP or DSA.

**Fig. 2:**
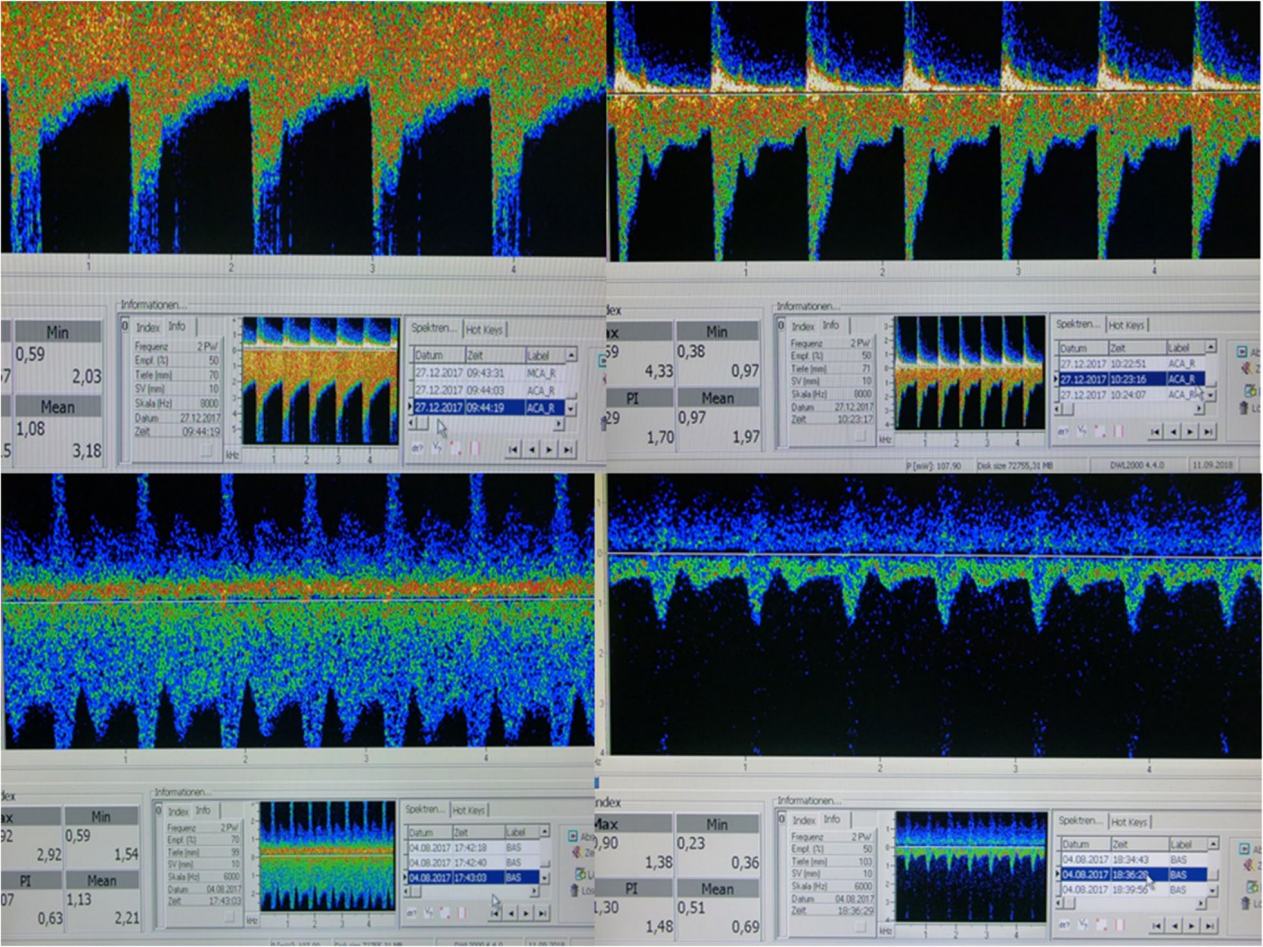
Transcranial Doppler ultrasound before and after sodium nitroprusside bolus. This figure depicts examples of transcranial Doppler sonography changes prompting an intraventricular administration of SNP bolus and its results. Upper panel: 48yo male, day 3 after subarachnoid hemorrhage, Glasgow Coma Scale-3, Hunt and Hess V, intracranial pressure 8 mmHg, mean arterial pressure 76 mmHg, Molsidomine 6 mg/h, no vasopressors. Left panel, 9:44 am: the right anterior cerebral artery prior to sodium nitroprusside; mean flow 318 cm/s. Right panel, 10:24 am: after 2 × 5 mg sodium nitroprusside, mean flow 197 cm/s, the clear shape of the signal. After the second bolus of sodium nitroprusside, 170 mg Urapidil. Lower panel: 56yo male, day 6 after subarachnoid hemorrhage, Glasgow Coma Scale-5, Hunt and Hess IV, intracranial pressure 17 mmHg, mean arterial pressure 97 mmHg, Molsidomine 16 mg/h, no vasopressors. Left panel, 5:43 pm: the basilar artery prior to sodium nitroprusside, mean flow 221 cm/s. Right panel, 6:36 pm: after 5 mg sodium nitroprusside, mean flow 69 cm/s. After sodium nitroprusside, 180 mg Urapidil

In 16/17 patients who achieved 16 mg/h of Molsidomine, severe vasospasm was documented in 380 complete TCDs (per patient mean 23; SD 21; range 4–86). In all, 208 TCD recordings revealed mean flow values exceeding 150–290 in anterior circulation or > 110 cm/s in BA, 172 TCDs documented broadening, mal-pulsatile and filiform (parvus or tardus) signals (assessed as an estimated flow of > 350 cm/s in the upstream stenotic segment). Each control TCD was performed soon after normalization of MAP and ICP elevations due to SNP administration to reduce the impact of “induced hypertension” or the natural course of vasospasm. Pathological findings substantially weakened or reversed in 96% of the cases instantly after single or repetitive boluses of SNP (mean dose 5.6 mg; SD 2.5 mg; range 2–10 mg). Broadening, filiform/parvus and tardus signals normalized either fully or almost completely within 30–90 min after SNP (Fig. 2, Table 2). On 51 occasions, 2–3 boluses (a total of 128 repetitions) with or without increasing SNP doses were required for TCD to return to normal flow profiles. One patient was a nonresponder to both Molsidomine and SNP.

**Table 2:** Changes in transcranial Doppler ultrasonography recordings in response to sodium nitroprusside intraventricular bolus

TCD findings: vasospasm and its resolution	MCA left	MCA right	ACA left	ACA right	VA/BA
Prior SNP: measurable values (no stump)	*n* = 70	*n* = 50	*n* = 42	*n* = 28	*n* = 18
Post-SNP: flow reduction mean (SD; range), *p* < 0.0001	− 40.4% (19.2%; 5–80%)	− 36% (17.0%; 5–90%)	− 40% (19.5%; 4–75%)	− 35% (15.7%; 5–66%)	− 48% (18.3%; 8–73%)
Post-first SNP: unchanged flow	*n* = 8; 11%	*n* = 3; 6%	*n* = 8; 20%	*n* = 4; 7%	*n* = 1; 5%
Prior SNP: stump or filiform signals	*n* = 25	*n* = 45	*n* = 25	*n* = 73	*n* = 4
Post-SNP: return to normalized signals *p* < 0.0001	*n* = 25	*n* = 45	*n* = 25	*n* = 63	*n* = 4

*ACA* anterior cerebral artery, *BA* basilar artery, *MCA* middle cerebral artery, *SNP* sodium nitro prusside, *SD* standard deviation, *TCD* transcranial doppler sonography, *VA* vertebral artery

In responders, the mean flow velocities decreased after single and repeated SNP administrations by 43% (mean 100 cm/s; SD 44 cm/s; range 40–300 cm/s; *p* < 0.0001) and 52% (mean 96 cm/s; SD 56 cm/s; range 40–350 cm/s; *p* < 0.0001).

The low-dose patient showed a relief of the spasms on TCD as well as a circulatory reaction (hypertension) from approximately 6 mg/h on, and we therefore assumed a threshold dose of Molsidomine would influence both spasms and systemic blood pressure by SNP.

After each SNP bolus, ICP increased immediately. A short-term rise in ICP < 30 mmHg was not addressed, whereas an increase of > 30 mmHg prompted intermittent CSF drainage until ICP returned to initial ranges, usually within < 5 min. Each bolus was carefully monitored for changes in MAP, which was kept at or below 105 mmHg. Systemic hypertension was often accompanied by tachycardia and evoked by an SNP bolus and lasted approximately 20–60 min; during that time, MAP, cranial perfusion pressure (CPP) and ICP were closely monitored. CPP changes were not documented due to rapid MAP changes, and ICP quickly returned to the initial value. No evidence for a Cushing reflex was observed.

Per TCD, a recurrence of a previously resolved vasospasm was often detected within 3–5 h after SNP bolus administration. Such progression, resolution and recurrence of vasospasm after SNP persisted for 8 days (SD 4 days; range 2–16 days).

**Fig. 3:**
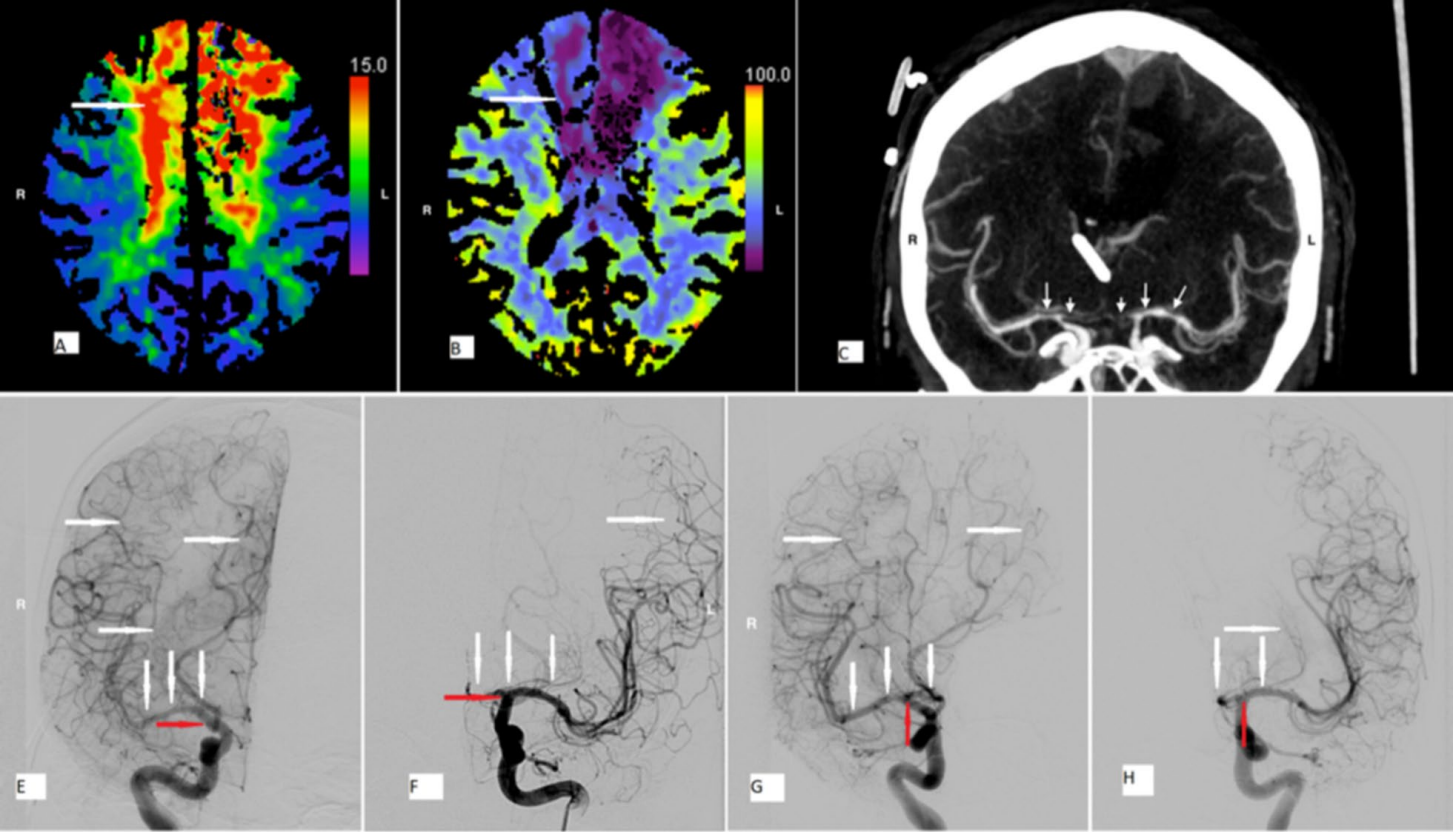
Sodium nitroprusside effect on cerebral perfusion and vasospasm in a patient after severe subarachnoid hemorrhage. Upper panel—CT perfusion **a** regional mean transit time and **b** cerebral blood flow during treatment interruption revealed high-grade perfusion disorder and brain at risk in the anterior cerebral artery territory (arrows). A brain infarction in the left anterior cerebral artery territory existed since clipping. **c** CT angiogram during molsidomine interruption confirmed transcranial Doppler sonography findings of the high-grade vasospasm in the anterior and the middle cerebral arteries. Arrows indicate vasospasm in both arteries. Lower panel—**e**–**h** Digital subtraction angiography after restarting molsidomine (1.5 h later; transcranial Doppler sonography was normal showing the anterior cerebral artery segment with no remaining perfusion gap and well filled the internal carotid and the middle cerebral artery. Left-sided aneurysm clipping induced truncation of the A2 segment of the anterior cerebral artery without perfusion disorder in the remaining territories. Arrows point at the full reversal of the high-grade spasm of the internal carotid artery at the bifurcation to the middle cerebral artery bilaterally and the anterior cerebral artery (right)

If imaging was performed before (CTA and CTP) and after (DSA) an application of SNP, the subsequent DSA showed the resolution of existing perfusion disorders and vasospasm (example: Fig. 3).

Moreover, after the introduction of a bridging scheme for nightly administrations of SNP without TCD guidance and during high-dose instances of Molsidomine administration, no more ischemic events occurred (*n* = 15 responding patients; 83%). No diffuse brain edema was observed.

The NIRS values after single and repeated SNP administrations increased by 17% (*p* < 0.0001) and 15% (*p* = 0.02), respectively (SD 14%; range 5–85%); these values remained unchanged in three patients (4%) and showed similar changes bilaterally, very probably indicating an augmentation of tissue oxygenation and suggesting a lack of a steal phenomenon.

The survival rate was 29.4% among responders. Twelve patients died, two because of reoccurrence of vasospasm at night and the remaining ten because of their critical illness or while within therapy limitations as per their living will. The nonresponder died of a massive stroke.

A follow-up performed by an independent physician (6 months after discharge) revealed that three patients had completely recovered and returned back to their former life and job (17.7%). One patient suffered left-sided, low-grade paresis but remained independent while working part-time (GOSE8/6; mRS0/1; NIHSS0/2; please see “Appendix”). No cognitive deficits occurred in any of these four patients (23.5%). One patient required high-grade care.

### Adverse Effects

In the context of Molsidomine, a rebound phenomenon can occur [29]. One patient developed a septic shock during the escalation of the Molsidomine dose, which required interruption for 24 h and high-dose vasopressors followed by its reintroduction at a low dose (1.6–3.6 mg/h) for 5 days. The patient rapidly developed severe vasospasm-associated brain infarctions after Molsidomine was stopped. Notably, despite the reinitiation of low-dose Molsidomine therapy, this patient did not respond with an expected MAP elevation to any SNP bolus (given every 4 h, *n* = 36) until the Molsidomine dose was > 6 mg/h, suggesting that a “saturation” dose of Molsidomine was necessary to exert a vasodilatory effect of SNP [18]. Afterward, no further infarctions occurred, and interestingly, each SNP bolus was accompanied by hypertension requiring an adequate response.

A second patient, who had an unintended interruption of Molsidomine infusion for > 3.5 half-lives, developed massive vasospasm with vessel truncation on CTA and CTP with critical perfusion disturbances (Fig. 3 and Table 3). After the urgent restarting of Molsidomine infusion and repeated SNP applications (*n* = 5; 6–10 mg), severe hypertension developed, and TCD values promptly normalized with DSA, revealing a dramatic improvement in perfusion. A next-day CT revealed small spotty infarctions in areas that formerly showed cerebral blood volume decreases. A rebound phenomenon is not expected with SNP boluses due to its very short half-life.

**Table 3:** Changes in imaging before and after sodium nitroprusside

Patient	TCD cm/s mean	CTA/CTP prior to SNP	Antihypertensive after SNP	SNP dose	DSA post-SNP
1	MCA L 290, ACA L stump	Mean transit time + time to peak prolonged in left MCA/ACA prolonged, no infarction	150 mg Urapidil	3 × 7 mg	Punctual stenosis in the left MCA (80%) and ACA 85%, ICA 60%, no remaining perfusion disturbance
2	MCA L 290, MCA R filiform, bilateral stump in the ACA	Massive prolongation of mean transit time + time to peak bifrontal, massive vasospasm with narrowing the MCA and the ACA about 95%	100 mg Propofol 300 mg Urapidil	3 × 6 + 8 + 10 mg and high-dose Molsidomine	MCA 30% narrowing, ACA R punctual 90%, ACA L 50% narrowing no new perfusion arrest
3	NA	Both MCA narrowing 70%, mean transit time + time to peak prolonged, CBF reduced in left MCA/ACA territories, no infarction	40 mg Urapidil 250 mg Propofol	2 × 5 mg + 7 mg SNP	No constriction > 50%, no perfusion disorder

These examples illustrate the relationship between transcranial Doppler ultrasonography and CT diagnostics (CT perfusion and CT angiography) before sodium nitroprusside application, the amount of antihypertensives required after sodium nitroprusside application to control evoked systemic hypertension and the subsequent alleviation of vasospasm on digital subtraction arteriography

2: After unintended Molsidomine interruption

*ACA* anterior cerebral artery, *CTA* CT angiography, *CTP* CT perfusion, *DSA* digital substraction angiography, *MCA* middle cerebral artery, *NA* not applicable, *SNP* sodium nitro prusside, *TCD* transcranial doppler sonography

In one patient, a preexisting normofrequent atrial fibrillation converted into an atrial tachyarrhythmia that was reversed by Metoprolol. Often, a sinus tachycardia emerged after SNP boluses. A single asymptomatic increase in methemoglobin (2.5%) occurred, and two patients received prophylactic sodium thiosulphate for cyanide poisoning. Vomiting and nausea were frequent. In CSF samples, one infection with *Staphylococcus aureus* was reported.

## Discussion

Despite the use of standard therapy, high Hunt–Hess grade (IV/V° w/wo pupil dilatation) patients have a heightened risk of DCIs, delayed brain infarctions (up to 65%) and poor outcomes (up to 90%) [30–32].

Overall, among all-grade surviving patients, DCI develops in 27–37%, 5–10% return to their former job [33] and 75% suffer from cognitive deficits [34].

Numerous therapeutic attempts have been used to address this serious prognosis. These include the use of SNP only as a bolus or continuously after aSAH. The results have been disappointing, inconsistent and unpredictable. Possible explanations are that these experiments mainly focused on spasms in large vessels and not on microcirculation at the parenchymal and cortical levels.

As the dilatation of these small vessels and the large brain arteries depend on the intravascular, intracerebral and paravasal availability of NO, we supplied exogenous NO (Molsidomine and SNP) to counteract the progressive loss of NO after aSAH.

In neurologically assessable patients, the trigger for the “local” administration of SNP was the development of DIND, which reversed promptly after SNP application.

Unfortunately, comatose patients elude the neurological examination required to initiate in-time intervention in cases of DIND. Moreover, previously, no perfect replacement of neurological monitoring was available because no monitoring device is perfect [36], and even continuous TCD or EEG recordings are prone to, for example, dislocation or misinterpretation. Unnoticed, perfusion disorders can develop at any time with the following infarcts. These patients can develop delayed ischemia unnoticed at any time after aSAH.

Being aware of this dilemma, we chose frequent TCD controls to obtain a certain overview of the possibly changing condition of each basal artery knowing that we could not obtain a reliable view of the microcirculation and parenchyma perfusion via TCD.

To schedule any necessary rescue interventions, the following monitoring criteria were measured: mean flow, the Lindegaard index and changes in the signal waveform (see above).

We also used bifrontal NIRS measurements to measure the general oxygenation of the tissue and any possible changes due to therapeutic procedures.

If a natural temporal window for ultrasound examination was not available, we drilled artificial windows on each side after obtaining consent from relatives.

Rapidly after SNP application, precarious pathological TCD findings weakened or reversed to a significant extent (*p* < 0.0001) and a significant increase (*p* < 0.0001) in tissue oxygenation was measured by the NIRS technique. Very likely, this indicates a success of the treatment, because (a) control measurements were performed after re-establishment of the baseline MAP and ICP, (b) a high-grade macrospasm persists despite the standard treatment and (c) pertinacious macrospasms extremely seldom resolve spontaneously in a rapid fashion. However, we cannot unequivocally exclude an impact of other variables than SNP and, therefore, further investigations are planned.

However, likely as a result of the use of abundant and repetitive TCD monitoring for the timing and control of bedside SNP application, the attenuation of spasms was fast enough to prevent a relevant number of secondary brain infarctions, thus reducing it to below the expected quantities in this subgroup (17% vs. 65%).

An analysis of therapy interruptions, based on the context of the available literature reporting on mono-therapy through SNP alone, revealed that presaturation with  ≥ 6 mg/h Molsidomine prior to the SNP bolus seems to be required to evoke an impact on TCD and NIRS values [12].

It could be a synergistic effect, which avoids the NO-sink effect [13], thereby supporting endothelial integrity and vascular reactivity and allowing SNP to exert its vasodilatory effect.

Because Molsidomine does not seem to have a clear influence on the spasms of the large vessels but instead appears to exert its effects on the microcirculation, a synergism may have developed from the combination of Molsidomine and SNP, which exerts its effect both on the macro- and in the microcirculation. This notion is supported by earlier reports, both experimental [14, 35] and clinical [17, 18], of the pathophysiological importance and clinical usefulness of NO-based therapy after aSAH.

Hypertension in response to SNP commonly occurs in combination with Molsidomine but is very rare with SNP alone. This systemic hypertension is believed to be a brainstem reflex to intracranial/intracerebral vasodilatation of spastic vessels [27] and should not be mistaken for a Cushing’s reflex that is accompanied by bradycardia. Additionally, ICP increases were promptly reversible, and no pupillary dilatation occurred.

It is very likely that some of the scheduled nightly SNP boluses delivered without TCD guidance were administered without vasospasm occurrence as we did not see major systemic hypertension after all applications. This observation supports the hypothesis that systemic hypertension was induced by effective vasodilatation of spastic vessels.

If these assumptions are correct, the surprising above-average outcome (17.7% returned to their former lives, 23.5% were able to work, and 29.4% survived) of some of our special patients who remained comatose long-term could be explained.

If so, TCD monitoring could be a useful strategy to achieve better-than-predicted outcomes in patients after aSAH and comatose patients because this may support the in-time use of rescue therapies to prevent the transition of reversible cerebral perfusion disorder into a permanent infarction.

We are presenting a small cohort, that was treated aside a standardized study condition, we are reporting on our clinical experience. A registered trial including sufficient numbers of patients with different World Federation of Neurosurgical Societies (WFNS)/Hunt and Hess grades will be necessary to validate our results. Fortunately, the German Government is going to sponsor a 3-armed multicenter study. Notably, hypertension evoked by SNP will preclude blinding during the treatment phase.

## Limitations

A small number of patients precluded randomization and a control group. Unfortunately, German law restricts the liberal use of uncommon compassionate or last resort therapies to only a few individuals and case-by-case decisions only. As mentioned above, a well-powered randomized study is planned.

Because we included the poorest grades in terms of the Hunt and Hess grading modified by Yasargil, selection bias and disease severity bias occurred. Furthermore, we compared our results with data obtained in roughly similar selected patients (these studies do not provide an exact description of patients’ conditions) in the literature, but definitions of gradings can vary (please see “Appendix”). However, an independent neuroradiologist analyzed the diagnostic imaging results, and follow-ups were performed by blinded independent physicians.

No measurement of serum or CSF levels of NO donors or associated products was possible due to technical limitations in our and associated laboratories. However, single and cumulating doses of both the Molsidomine and SNP used in our study are common in clinical practice (Molsidomine) or in the literature (SNP). Molsidomine amounts reached the highest recommended dosage in cardiac surgery, and SNP doses were at a medium range compared to the literature. Of notice, the rebound phenomenon that can occur during the use of Molsidomine strictly needs to be avoided.

For rescue therapy, we used intra-arterial vasospasmolysis also—this could be a confounder. However, to date, a benefit for patients treated with this therapy has not been proven and could be described as poor, especially in cases with high Hunt and Hess/WFNS gradings [37].

We could not address possible SNP-related neurotoxicity. However, in a different case series in which SNP was applied continuously, reaching total amounts of 200 mg/24 h, no toxicity was reported. (The maximal dose of SNP used in our study was 76 mg/24 h.)

## Conclusions

In long-term intubated comatose patients with or without pupil dilation after severe aSAH, dual-compartment administration of two NO donors seems to be feasible and safe. Most likely, combined NO donors promptly as well as reliably led to a significant reduction in high-grade macrospasm and a reciprocal rise in tissue oxygenation as measured by TCD and NIRS prior and after SNP application. However, seldom a rapid resolution of high-grade macrospasm could occur either in the spontaneous course or by standard emergency treatment.

Frequent TCD evaluation detected critical changes in TCD flow velocities and shapes that promptly induced the bedside application of SNP without loss of time due to transportation, preparation of other rescue treatments or diagnostics.

It is likely that SNP diminished macrospasms before the development of irreversible brain infarctions evolved. The planned nocturnal SNP boluses were delivered on a fixed time schedule and were carried out by the doctors on duty in the NICU, and this probably also contributed to the reduction in relevant vasospasms and perfusion disorders. This instantly applicable bedside tool for the NICU team could prevent severely affected patients from having to undergo transport, examinations and emergency treatments, which are dangerous and costly in terms of patients and personnel but have not been proven effective.

In our small cohort, we observed much lower rates than expected of delayed brain infarctions in long-term comatose patients, and this perhaps led to better-than-estimated outcomes. Obviously, an evaluation of our preliminary results is needed. The German government is going to sponsor a prospective, randomized, statistically sufficiently powered study.

## Appendix

### NO in the Context of SAH

Hemoglobin degradation is probably the most important factor that leads to nitric oxide (NO) deficiency, but other factors may also induce NO shortage via additional pathomechanisms (transient ischemia, reactive oxygen species (ROS) production, etc.) evoked by aneurysmal subarachnoid hemorrhage (aSAH).

To understand the NO hemoglobin/cerebral blood flow (CBF) relationship after aSAH, it is important to recognize that 70% of regional (rCBF) and total CBF [38] are regulated by microcirculation, which is strictly dependent on the local presence of NO [6, 14]. In turn, only 30% of rCBF is contributed by major arterial vessels. Therefore, it has been concluded that brain ischemia can easily occur without evidence of visible spasm in conductive arteries and that, in turn, if microcirculation remains patent, a measurable spasm will not necessarily lead to a cerebral infarction [15, 39, 40]. The relevance of microcirculatory disorders has also been supported by the finding that the complete elimination of detectable spasms in the macrocirculation by a selective endothelin receptor antagonist (clazosentan) did not improve outcomes or the delayed cerebral ischemia (DCI) rate and decreased the rate of vasospasm-associated infarcts only marginally [41, 42]. Moreover, brain vessels feature a basal constrictive tone (39) that must be continuously counteracted by NO. Consequently, a lack of NO affects the CBF at both the micro- and macro-level after aSAH [4, 7, 13].

Aside from vasoregulatory tasks, NO also regulates platelet activity, which explains why NO deficiency can lead to microembolism, particularly in (cortical) end vessels. This mechanism has been suggested as a possible cause of thrombosis and infarctions [43, 44].

*Molsidomine* is a NO donor with unique and favorable pharmacodynamic and pharmacokinetic profile characteristics. The active component of Molsidomine is SIN-1, which is cleaved in the liver. The release of NO from SIN-1 takes place by spontaneous decomposition; the metabolites are pharmacologically inactive, and when applied at a clinical dose, SIN-1 does not display toxicity, has no known relevant plasma protein binding ability or associated interactions, and does not accumulate. Molsidomine has a linear dose–effect and a large therapeutic dose range and has been used in the treatment of coronary heart diseases for the prophylaxis of coronary spasms for several years. Moreover, its use does not lead to any relevant compromise of systemic hemodynamics. It can be applied intravenously and does not evoke tolerance. Dosage adjustment is not required in cases with liver or kidney impairment or congestive heart failure [45–48].

The main effect of SIN-1 is to increase the intracellular cyclic guanosine monophosphate concentration. This induces the relaxation of arterioles and large venous and arterial vessels independent of the endothelium, a decrease in the tone of smooth vascular muscles, the concentration-dependent inhibition of platelet aggregation, and the release of platelet-derived vasoactive compounds. SIN-1 increases nitrite/nitrate levels and inhibits adenosine, thrombin, serotonin, collagen and “platelet-activating factor” [45, 46, 49].
